# Comprehensive analysis of the expression profiles of mRNA, lncRNA, circRNA, and miRNA in primary hair follicles of coarse sheep fetal skin

**DOI:** 10.1186/s12864-024-10427-7

**Published:** 2024-06-07

**Authors:** Dehong Tian, Quanbang Pei, Hanjing Jiang, Jijun Guo, Xianghua Ma, Buying Han, Xue Li, Kai Zhao

**Affiliations:** 1 Qinghai Provincial Key Laboratory of Animal Ecological Genomics, Key Laboratory of Adaptation and Evolution of Plateau Biota, Northwest Institute of Plateau Biology, Chinese Academy of Sciences, Xining, 810000 Qinghai China; 2 University of Chinese Academy of Sciences, Beijing, 100049 China; 3 Qinghai Sheep Breeding and Promotion Service Center, Gangcha, 812300 Qinghai China; 4 Qinghai Livestock and Poultry Genetic Resources Protection and Utilization Center, Xining, 810000 Qinghai China; 5 General Station of Animal Husbandry of Qinghai Province, Xining , 810000 Qinghai China; 6 Hainan Tibetan Autonomous Prefecture science and technology extension service center, Hainan Tibetan Autonomous Prefecture, Qinghai, 813000 China

**Keywords:** Tibetan sheep, Hair follicle development, Transcriptome, ceRNA

## Abstract

**Background:**

The Qinghai Tibetan sheep, a local breed renowned for its long hair, has experienced significant deterioration in wool characteristics due to the absence of systematic breeding practices. Therefore, it is imperative to investigate the molecular mechanisms underlying follicle development in order to genetically enhance wool-related traits and safeguard the sustainable utilization of valuable germplasm resources. However, our understanding of the regulatory roles played by coding and non-coding RNAs in hair follicle development remains largely elusive.

**Results:**

A total of 20,874 mRNAs, 25,831 circRNAs, 4087 lncRNAs, and 794 miRNAs were annotated. Among them, we identified 58 DE lncRNAs, 325 DE circRNAs, 924 DE mRNAs, and 228 DE miRNAs during the development of medullary primary hair follicle development. GO and KEGG functional enrichment analyses revealed that the JAK-STAT, TGF-β, Hedgehog, PPAR, cGMP-PKG signaling pathway play crucial roles in regulating fibroblast and epithelial development during skin and hair follicle induction. Furthermore, the interactive network analysis additionally identified several crucial mRNA, circRNA, and lncRNA molecules associated with the process of primary hair follicle development. Ultimately, by investigating DEmir’s role in the ceRNA regulatory network mechanism, we identified 113 circRNA–miRNA pairs and 14 miRNA–mRNA pairs, including IGF2BP1-miR-23-x-novel-circ-01998-MSTRG.7111.3, DPT-miR-370-y-novel-circ-005802-MSTRG.14857.1 and TSPEAR-oar-miR-370-3p-novel-circ-005802- MSTRG.10527.1.

**Conclusions:**

Our study offers novel insights into the distinct expression patterns of various transcription types during hair follicle morphogenesis, establishing a solid foundation for unraveling the molecular mechanisms that drive hair development and providing a scientific basis for selectively breeding desirable wool-related traits in this specific breed.

**Supplementary Information:**

The online version contains supplementary material available at 10.1186/s12864-024-10427-7.

## Background

The wool fibers of Tibetan sheep, derived from primary hair follicles, primarily consist of myeloid wool, making carpet wool a valuable genetic resource known for its high quality. There is currently widespread interest in the study of hair follicle development in sheep and awareness of its importance for understanding the adult fleece and controlling wool production [1]. The initial stage of hair follicle development is a complex process, commencing with the proliferation of epidermal cells to form a placode beneath which an aggregation of dermal cells occurs, and these two cell populations interact and undergo orderly proliferation and differentiation. Subsequently, clumps gradually emerge within the dermis, ultimately giving rise to primary hair follicles, while certain newly formed hair follicles progress into secondary ones [2]. The rapidly proliferating matrix cells in the hair bulb produce hair shafts, and the dermal papilla consists of specialized fibroblasts located at the base of the hair follicle, which control the number of matrix cells and thus the size of the hair [3]. Primary hair follicle is characterized by the presence of large sebaceous glands and sweat glands, with both the diameter of hair follicles and hair bulbs being larger than those of secondary hair follicles, which develop earlier. However, all types of hair follicles possess complete sebaceous glands that contain a certain proportion of hollow or myelinated rough and elastic hairs [2, 4]. The morphology structure and development of hair follicles have important effects on the economic performance of wool. Studying the molecular mechanisms of sheep hair follicle morphogenesis can provide a refined and highly instructive research model for addressing key problems in modern biology [2], and will also contribute to understanding the genetic basis of wool related traits.

As the complexity of trait analysis increases, non-protein-coding sequences are increasingly prevalent in the genomes of multicellular organisms. Various classes of small and large non-coding RNAs (ncRNAs) have been demonstrated to regulate gene expression at almost every level, encompassing chromosome dynamics, chromatin architecture, transcriptional control, post-transcriptional processing, and translation, focusing primarily on animals [5]. Research over the last two decades revealed new classes of ncRNAs, including microRNAs (miRNAs), long ncRNAs (lncRNAs), and circular RNAs (circRNAs), all with different regulatory functions that are interwoven together in the larger RNA communication network to regulate the fundamental protein effectors of cellular function [6], including the regulation of hair follicle morphogenesis and development through DNA-binding transcriptional regulators, transcriptional activation, RNA editing, RNA interference and translational regulation [7–10]. Recent studies have shown that RNAs influence each other’s levels by competing for a limited pool of microRNAs, giving rise to a new theory of “competitive endogenous RNA” (ceRNA). mRNAs, lncRNAs, miRNAs, and circRNA activities form a large-scale regulatory network across the transcriptome, greatly expanding the functional genetic information in the genome and playing an important role [11]. Genome-wide identification of mRNAs, lncRNAs, circRNAs and miRNAs can take significant effect in processes of bovine intra-muscular adipogenesis [12]. circRNA-miRNA-mRNA ceRNA regulatory network affects muscle growth and development and individual growth by regulating the transformation of oxidized muscle fibers and glycolytic muscle fibers in Tibetan sheep [13].

Several key signals in WNT, BMP, EDAR, and FGF pathways, and a series of lncRNAs, were shown to be potentially important roles of in primary hair follicle induction and skin development [14]. More and more new evidence suggests the existence of a large network of regulatory ncRNAs, which constitute the sequence programming regulatory information of biological organisms and play an important role in the epigenetic development of their traits. Moreover, the variation of complex characteristic sequences primarily resides in the non-coding region, presumably the regulatory region, which is likely to alter the interaction between regulatory RNAs and their target, a prospect needs to be taken into consideration [15]. However, there are few reports about the transformation mechanism of hair follicle and skin development underlying regulation of the ceRNA network in carpet sheep fetal skin at placode stages.

To investigate the key genes, signaling pathways, and regulatory networks involved in medullary wool morphogenesis, our focus was directed towards analyzing the gene expression profiles of primary and secondary hair follicles. We utilized RNA-seq technology to investigate the regulatory role of competing endogenous RNAs (ceRNA) in the development of hair follicles in sheep skin and constructed a ceRNA regulatory network to identify key factors involved in hair follicle formation. This study will serve as a foundation for further research on hair follicles, provide insights into the interaction networks of ceRNA during the initiation of primary hair follicles, and provide a scientific basis for the breeding of wool-related traits.

## Materials and methods

### Animals and samples preparation

Carpet wool Tibetan sheep from Qinghai sheep breeding and promotion service center were used in this study. The study selected six healthy Tibetan ewes (3 years old; mean fiber diameter of coarse wool, 58.98 ± 2.48 μm; cashmere wool, 18.71 ± 2.73 μm) that did not exhibit estrus during the second estrus period following artificial assisted mating with the same ram, and these ewes were maintained under identical conditions. Based on previous morphological studies on the genesis and development of hair follicles [2], fetal skin samples were collected in pregnant ewes at E65 (TWP) and E85 (TWS). These samples were immediately placed in liquid nitrogen (− 80 ℃) for the transcriptome sequencing and Real-time quantitative polymerase chain reaction analysis (RT-qPCR).

All skin tissue (diameter 2 cm) was collected from the right medial area behind the shoulder blade to make a section and rinsed with 1 x phosphate buffered saline (PBS), then prepared paraffin-embedded sections and fixed in 4% paraformaldehyde at 4 °C for approximately one week before H&E staining.

### RNA extraction, library construction

Total RNA was extracted using the Trizol reagent kit (Invitrogen, Carlsbad, CA, USA) according to the manufacturer’s protocol. The quality and quantity of RNA were verified with 1% agarose gel, a NanoPhotometer® spectrophotometer (Implen, USA), Qubit® 2.0 Fluorometer (Life Technologies, USA), and Agilent Bioanalyzer 2100 system (Agilent Technologies, Palo Alto, CA, USA). A total of 1.5 µ g RNA was used for sequencing library construction. The RNA-seq libraries were constructed using the NEBNext® Ultra™ RNA Library Prep Kit for Illumina® (NEB, USA) utilizing the manufacturer’s instruction. After purification and end-repair, the poly-A tail and sequencing adapters were ligated. Ligation products were size-selected by agarose gel electrophoresis, PCR amplified, and sequenced using Illumina HiSeq4000 by Gene Denovo Biotechnology Co (Guangzhou, China).

### circRNA, lncRNA and miRNA library construction and sequencing

Fastp [16] (version 0.18.0) was used to filter adapters or low quality bases from raw reads. Short reads alignment tool Bowtie2 [17] (version 2.2.8) was used for mapping reads to ribosome RNA (rRNA) database. The rRNA removed reads of each sample were then mapped to reference genome (ncbi_GCA_002742125.1_Oar_v1.0) by HISAT2 [18].

Identification of circRNA: 20mers from both ends of the unmapped reads were extracted and aligned to the reference genome to find unique anchor positions within splice site. Anchor reads that aligned in the reversed orientation (head-totail) indicated circ RNA splicing and then were subjected to find_circ to identify circ RNAs [19].

Identification of lncRNA: All reconstructed transcripts were aligned to reference genomes to obtain new transcripts. We used the following parameters to identify reliable novel genes: the length of transcript was longer than 200 bp and the exon number was more than two. Three softwares CNCI [20] (version 2) and CPC [21] (version 0.9-r2) and FEELNC [22] (version v0.2) were used to choose long non-coding RNAs.

Identification of miRNA: All of the clean tags were aligned with small RNAs in GeneBank database (Release 209.0) to identify and remove rRNA, scRNA, snoRNA, snRNA and tRNA. All of the clean tags were then searched against miRBase database (Release 22) to identify known miRNAs. The known miRNAs were identified by comparison with miRNAs alignment from other species. All of the unannotated tags were aligned with reference genome. According to their genome positions and hairpin structures predicted by parameters of software mirdeep2, the novel miRNA candidates were identified.

### Differentially expressed RNAs

To identify differentially expressed (DE) lncRNAs, mRNAs, circRNAs and miRNAs across three biological replicates samples, the edgeR package software between two different groups was used [23]. The fragments per kilobase per million mapped fragments (FPKM) value was calculated to quantify expression abundance of lncRNA and mRNA, the reads per million mapped reads (RPM) were used to compute expression abundance of the identified circRNAs, and the miRNA expression level was calculated and normalized to transcripts per million (TPM). Significant differential expression lncRNA and mRNA were identified with |log2Ratio| ≥ 1 and qval < 0.05 as screening conditions for significant DE genes. For circRNAs, fold change ≥ 2 and pval < 0.05 were used as screening parameter for significant DE circRNAs. miRNAs were identified with pval ≤ 0.05, and fold change ≥ 2 in a comparison as significant DE miRNAs.

### RT-qPCR analysis

Several differentially gene expressed level involved in primary hair follicle induction were selected and confirmed by RT-qPCR. The housekeeping gene GAPDH and actin were used to normalize the expression levels of the lncRNAs, circRNAs and mRNAs, and U6 was used to normalize the expression levels of miRNAs. The primer sequences of the selected lncRNAs, mRNAs, circRNAs and miRNAs are listed in Supplementary Table 1. Quantitative real-time PCR was carried out in a 20 µL reaction mixture containing 10 µL 2×Top Green EX-Taq Mix, 2 µL cDNA, 7 µL ddH_2_O, and 0.5 µL forward and reverse primers. The following thermocycling program was used: 1 cycle of 94 ℃ for 30 s; 42 cycles of 94 ℃ for 5 s, 61 ℃ for 35 s; 1 cycle of 95 ℃ for 10 s, 65 ℃ for 60 s, 97 ℃ for 1 s followed by a final melting curve analysis stage. The relative expression levels were calculated using the 2^−ΔΔ CT^ method [24]. All experiments were performed in three biological replicates.

### GO and KEGG functional enrichment analysis

Gene Ontology (GO) enrichment and Kyoto Encyclopedia of Genes and Genomes (KEGG) pathway analysis were analyzed using the Gene Ontology database (http://www.geneontology.org/), and KEGG was the major public pathway-related database (http://www.kegg.jp/kegg/). The calculating formula of *P*-value is: $\text{P}=1-\sum _{i=0}^{m-1}\frac{\left(\frac{M}{i}\right)\left(\frac{N-M}{n-i}\right)}{\left(\frac{N}{n}\right)}$, here N is the number of all genes with GO/KEGG annotation; n is the number of source genes in N; M is the number of all genes that are annotated to the certain GO terms / specific pathways; m is the number of source genes in M.

### Interaction Network Construction

For prediction of mRNAs interacting with circRNAs, lncRNAs and miRNAs, miRTarBase [25] (version 6.1) was used to predict mRNAs targeted by miRNAs sponge. The resulting correlation of circRNAs-miRNAs-lncRNAs-mRNAs can be visualized by Cytoscape [26] software to present a core and hub gene biological interact. The construction of ceRNA regulatory network complies with the following three aspects : (1) co-expressed negatively mRNA-miRNA or lncRNA / circRNA-miRNA expression correlation pairs was chosen using the Spearman Rank correlation coefficient (SCC) < -0.7. (2) co-expressed lncRNA–mRNA pairs or circRNA-mRNA pairs were selected by the PCC > 0.9. (3) Hypergeometric cumulative distribution function test was selected to test common miRNA sponges between two genes with *p* values <0.05. The calculating formula of *P*-value is: $\text{p v}\text{a}\text{l}\text{u}\text{e}=1-\text{F}\left(x/U,M,N\right)=1-\sum_{i=0}^{x-1}\frac{\left(\frac Mi\right)\left(\frac{U-M}{N-i}\right)}{\left(\frac UN\right)}$.

## Results

### Histological characteristics of medullary hair follicles during development

**Fig. 1:**
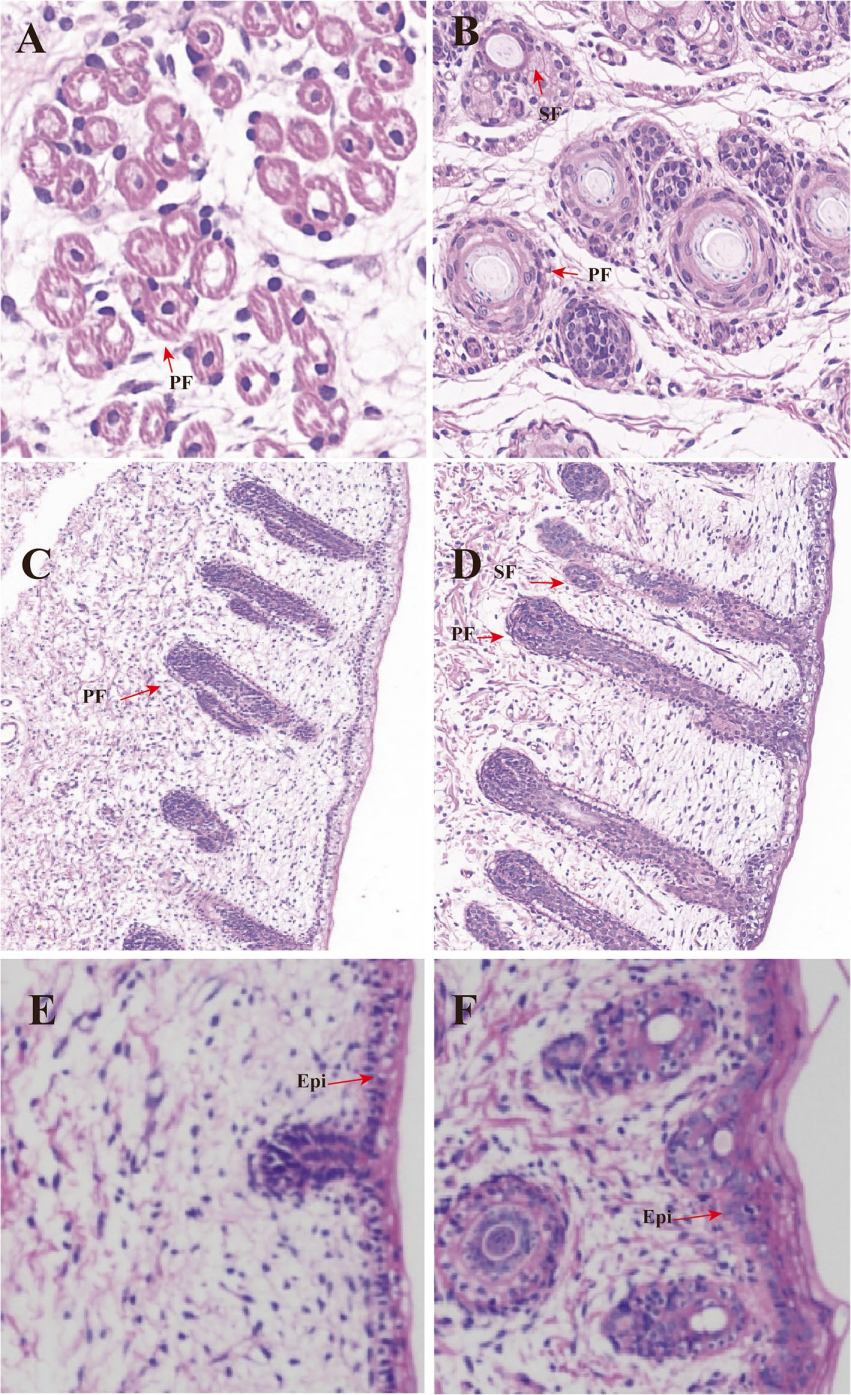
Hematoxylin-eosin (HE) staining of sheep hair follicles at two developmental stages. Horizontal (magnification: ×400) (**A**, **B**) and longitudinal (×400) (**C**, **D**) slices of skin of primary (**A**, **C**) and secondary hair follicles (**B**, **D**). Horizontal (×100) and longitudinal (×100) slices of skin of primary hair follicle (**E**, **F**). PF: Primary hair follicle; SF: Secondary hair follicle; Epi, epidermis

Histological changes in early hair follicle development can be observed in horizontal and longitudinal skin sections stained with the haematoxylin-eosin (H&E). The formation of primary hair follicles (PFs) is indicated by the emergence of hair placode and dermal condensate at E65, while the sheep fetal skin epidermis appears thin and homogeneous (Fig. 1A, C and E). The formation of secondary follicles (SFs) commenced at E85, with PFs exhibiting earlier development. Additionally, PFs displayed increased size and length, while SFs appeared smaller and grew in close proximity to the PFs epidermis. Furthermore, the dermis of sheep skin exhibited greater thickness compared to the preceding period (Fig. 1B, D and F).

### Overview of mRNAs/circRNAs/lncRNAs sequencing

**Fig. 2:**
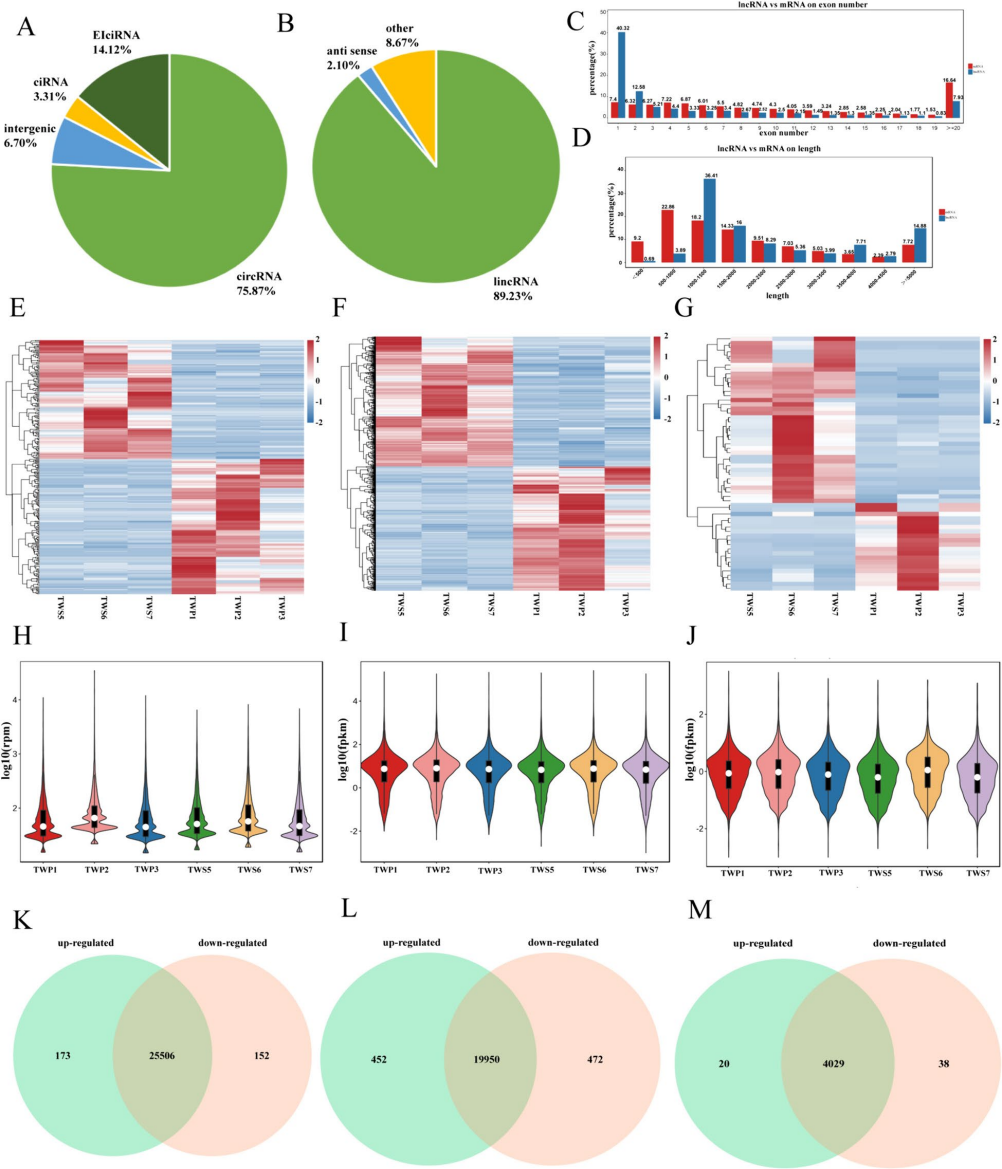
Expression patterns of mRNAs, circRNAs and lncRNAs. **A** The type and proportion of circRNAs. **B** The type and proportion of lncRNAs. **C** Exon number distribution of lncRNAs and mRNAs. **D** Transcript lengths distribution of lncRNAs and mRNAs. **E**-**G**. Clustered expression heatmaps of all mRNAs. **H**-**J**. Violin plot of gene expression levels all mRNAs. **K-M**. The Venn diagrams of the shared and unique differential expressed circRNAs, mRNAs, lncRNAs

After filtering out the low-quality reads, a total of 520,980,026 valid reads with Q30 greater than 91.46%were obtained in all samples, of which more than 88.99% were mapped to the reference genome of *Ovis aries* (Supplementary Table 1). A total of 20,874 mRNAs (novel-genes 373), 4087 lncRNAs (3315 existing and 772 novel), and 25,831 circRNAs with an average length of 891 bp as novel circRNAs were identified during medullary hair follicle development (Supplementary Table 2). Meanwhile, the identified lncRNAs contained 89.23% long intergenic noncoding RNA (lincRNAs), and 75.87% of typical circRNAs were identified (Fig. 2A, B). The length distribution of lncRNAs was consistent with that of protein-coding gene. Both lncRNAs and mRNAs contain exons from 1 to 20, most lncRNAs had 1–2 exons. (Fig. 2C). The distributions of lncRNAs and mRNAs lengths were consistent (Fig. 2D). The expression heatmaps of circRNAs, mRNAs, lncRNAs showed inter-group and intra-group differences and clustering differentiation (Fig. 2E, F, G). In addition, the violin plot showed differences in circRNA, mRNA, lncRNA expression distribution (Fig. 2H, I, J). Two groups showed a total of significantly differentially expressed 325 DE circRNAs (including significantly 173 up-regulated and 152 down-regulated), 299 circRNAs were derived from source genes, and residual 26 circRNAs were derived from intergenic regions, having no source genes, 924 DE mRNAs (including significantly 452 up-regulated and 472 down-regulated), and 58 DE lncRNAs (including significantly 20 up-regulated and 38 down-regulated) (Fig. 2K, L, M).

### Differential expression analysis of mRNA/circRNA/lncRNA

**Fig. 3:**
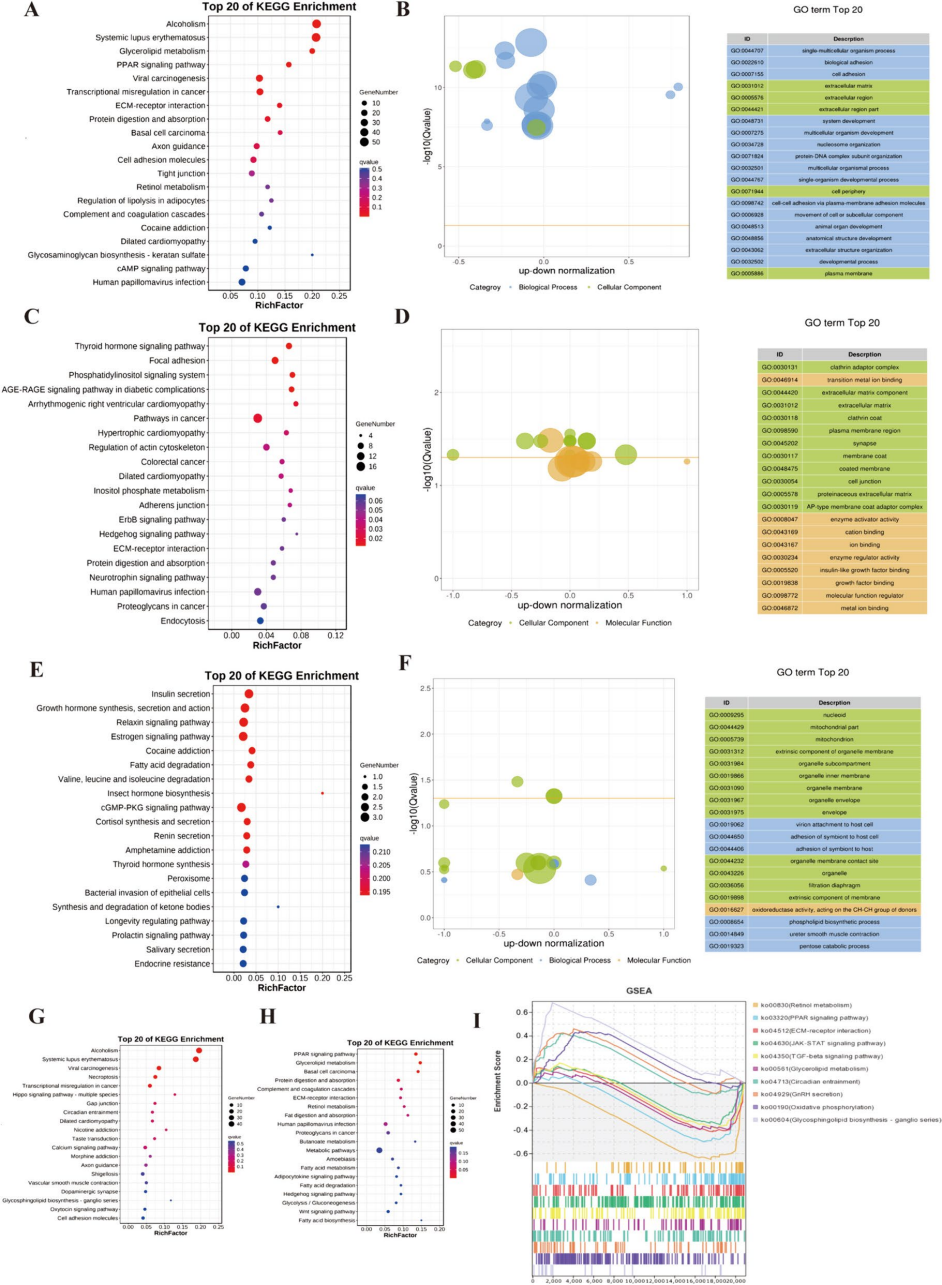
GO functional annotation and KEGG enrichment analysis. **A-B**. The top 20 KEGG and GO enrichment pathways for DE mRNAs. **C-D**. The top 20 KEGG and GO enrichment pathways for DE circRNAs. **E-F**. The top 20 KEGG and GO enrichment pathways for DE lncRNAs. **G-H**. Pathways enriched by up-regulated or down-regulated DE mRNAs. **I**. GSEA enrichment analysis of selected results

To discover the biological functions of differentially expressed genes (DEGs) during early hair follicle development, functional enrichment analysis of stage-specific DEMs, host genes of DECs and cis-regulated genes of DELs were performed. The results showed that the GO terms and KEGG signaling pathways of DEMs were mainly involved in PPAR signaling pathway, ECM-receptor interaction, Retinol metabolism, cAMP signaling pathway, biological adhesion, and cell adhesion (Fig. 3A and B, Supplementary Tables 3 and 4). In addition, we found that some pathways were significantly enriched in up-regulated and down-regulated genes, respectively (Fig. 3G and H, Supplementary Tables 5 and 6), after further GSEA analysis of these pathways by Dex treatment, we found that the Circadian entrainment and Glycosphingolipid biosynthesis pathways were significantly activated, the PPAR signaling pathways, ECM receptor interaction, and Retinol metabolism were suppressed (Fig. 3I). These pathways were highly related to the early stage of hair follicle induction. DECs might be mainly enriched in Phosphatidylinositol signaling system, Hedgehog signaling pathway, ECM-receptor interaction, clathrin coat and growth factor binding (Fig. 3C and D, Supplementary Tables 7 and 8). The results of functional enrichment analysis of DELs indicated that DELs might be involved in cGMP-PKG signaling pathway, Prolactin signaling pathway and phospholipid biosynthetic process (Fig. 3E and F, Supplementary Tables 9 and 10).

### Construction of the DE circRNA/lncRNA–mRNA interaction network

**Fig. 4:**
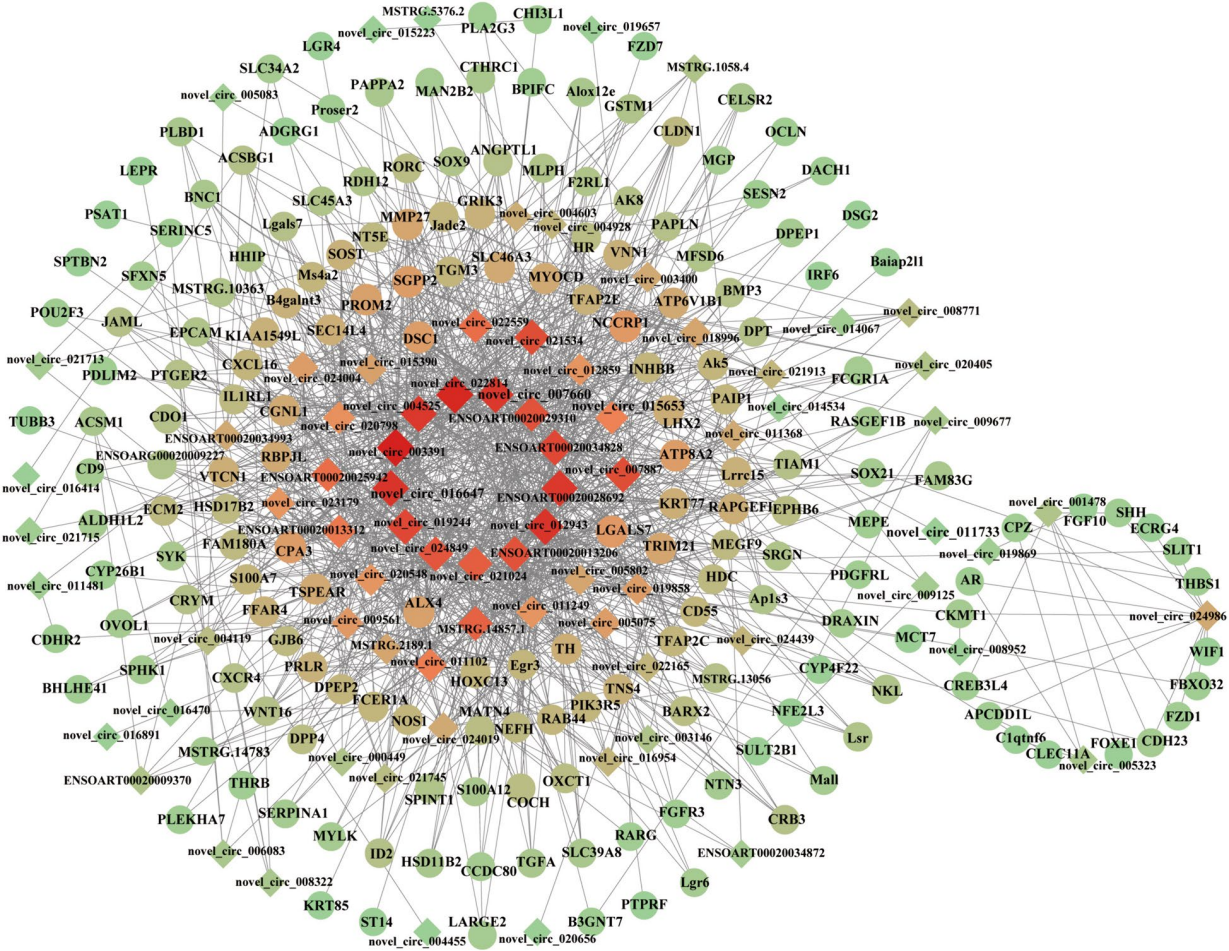
circRNA/lncRNA-mRNA interaction network. The edges are the Pearson correlation coefficient (PCC) between genes. DE mRNAs and DE circRNAs / lncRNAs are represented by ellipse and diamonds, respectively

To further elucidate the key genes involved in primary hair follicle development, we subsequently constructed a circRNA/lncRNA-mRNA network interaction map based on the identified shared differential genes (Fig. 4, Supplementary Table 11). A total of 64 potential DECs and 13 key DELs that are likely to play crucial roles in the development of primary hair follicles. The findings revealed that the interaction network comprised a total of 1056 connections, with each lncRNA exhibiting associations with multiple mRNAs. A total of 12 circRNAs and three lncRNAs were identified as co-expressed with Dsc1, while 13 circRNAs and one lncRNAs were found to exhibit co-expression with ALX4, the network also includes circRNAs/lncRNAs that are co-expressed with Egr3, TGM3, KRT77, HOXC13, TSPEAR, MEGF9, SHH, SOX9, PTGER2. The aforementioned circRNAs/lncRNAs associated with genes may exert a significant influence on the regulation of hair follicle formation and growth.

### Identification of a miRNA landscape

**Fig. 5:**
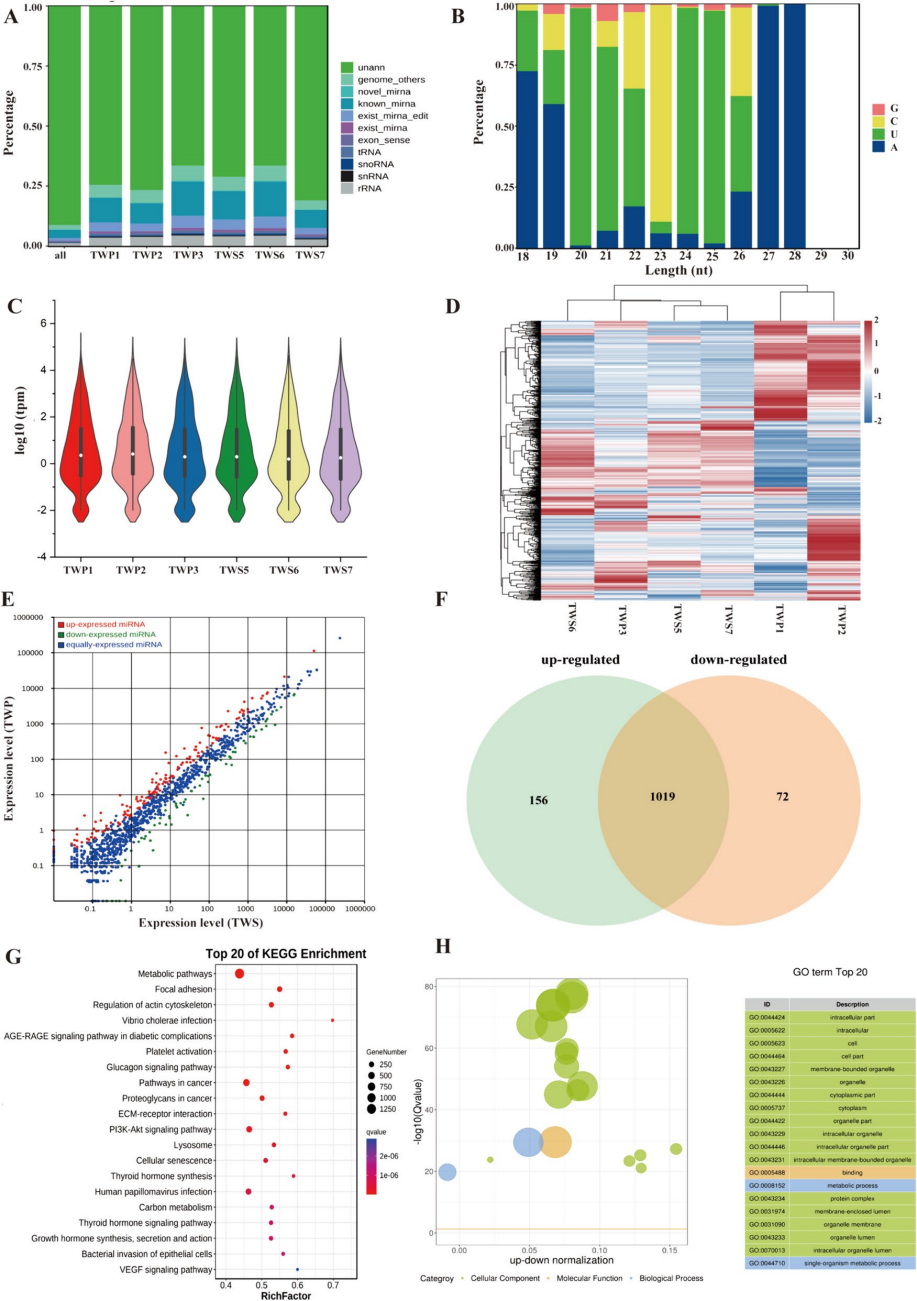
The landscape of miRNA. **A** The tag type of miRNAs. **B** First base preference of different tag lengths. **C** Violin plot of miRNAs expression levels. **D** Clustered expression heatmap of all miRNAs. **E** Scatter plots of all miRNAs. Red represents up-expressed miRNAs. Green represents down- expressed miRNAs. Blue is equally-expressed miRNAs. **F** Venn diagram showing the up-regulated and down-regulated DE miRNAs. **G-H**. The top 20 KEGG and GO enrichment pathways analysis of DE miRNAs

By removing other non-coding RNAs such as rRNA, scRNA and tRNA, a total of 794 miRNAs were obtained (Fig. 5A). The initial base of these miRNAs was uridine and they were mostly expressed at the middle (0.01 < percentage < 1) levels (Fig. 5B). Violin plot and clustered expression heatmap of miRNA showed expression distribution differences and clustering differentiation (Fig. 5C and D). There were 156 upregulated and 72 downregulated DE miRNA that were identified (Fig. 5E and F). The biological functions of the target genes of these DE miRNAs were further analyzed by functional enrichment. The GO terms and KEGG signaling pathways were mainly involved in ECM-receptor interaction, PI3K-Akt signaling pathway, Growth hormone synthesis, secretion and action, VEGF signaling pathway, metabolic process, intracellular organelle (Fig. 5G and H, Supplementary Tables 12 and 13).

### Construction of the DE circRNA/lncRNA–miRNA–mRNA ceRNA regulatory network

**Fig. 6:**
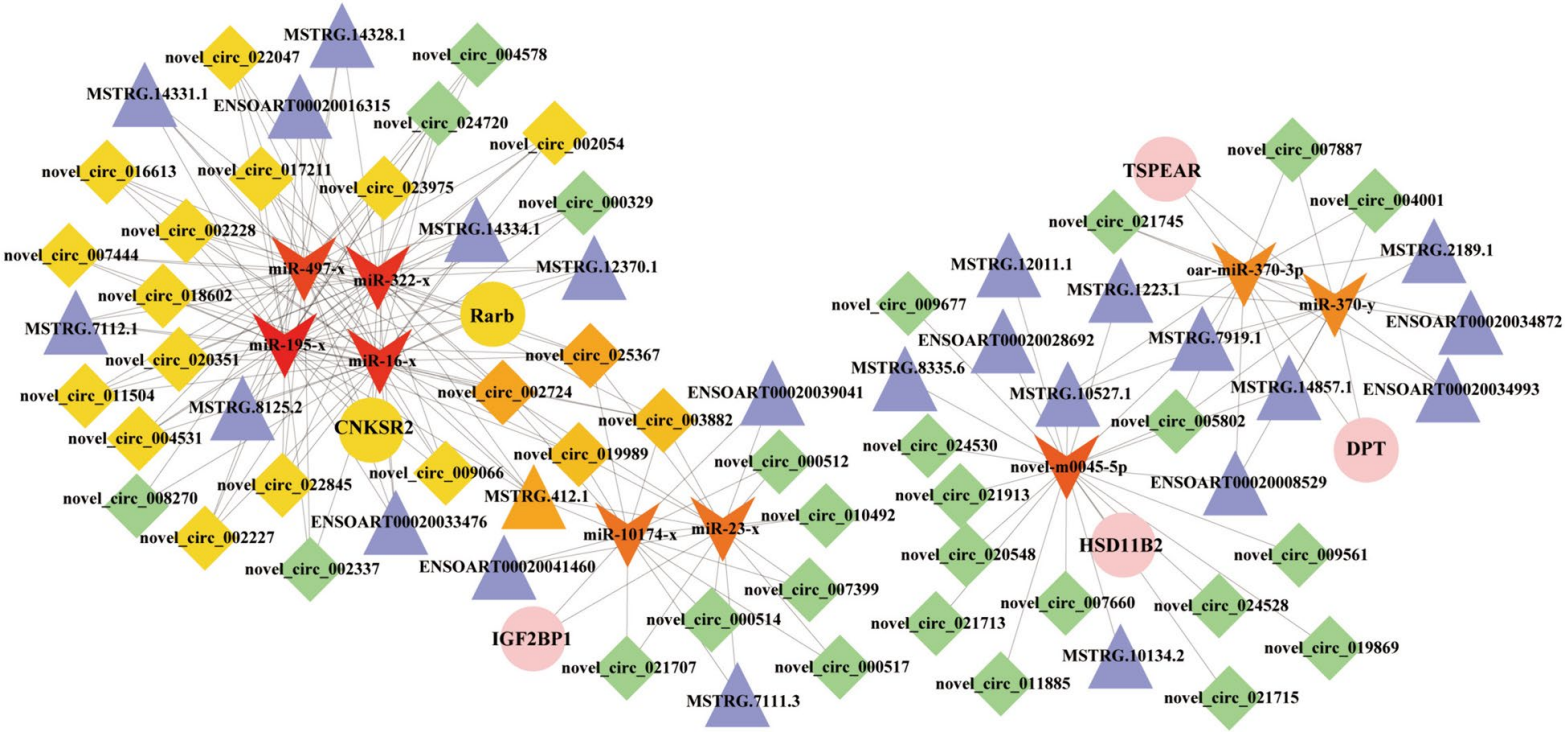
The ceRNA co-regulation network. The circle, triangle, diamonds, and V-type represented the DE mRNAs, DE lncRNAs, DE circRNAs, and DE miRNAs. The higher the connectivity, the redder the color

The regulatory network of circRNA/lncRNA-mRNA interaction integrates the prediction results of miRNA target genes, thereby facilitating the exploration of regulatory elements governing primary hair follicles. The participation of DEmiRs in the ceRNA regulatory network was analyzed, and subsequently integrated with the circRNA/lncRNA-mRNA regulatory relationship to construct a comprehensive ceRNA network (Fig. 6, Supplementary Table 14). The ceRNA regulatory network contained 113 circRNA–miRNA pairs and 14 miRNA–mRNA pairs and included 6 mRNAs, 44 circRNAs, 24 lncRNAs and 9 miRNAs. MSTRG.7111.3, novel-circ-019989 and miR-23-x have shared target gene IGF2BP1, Similarly, we observed the same findings in DPT-miR-370-y-novel-circ-005802-MSTRG.14857.1 and TSPEAR-oar-miR-370-3p-novel-circ-005802-MSTRG.10527.1. The network regulation also involves HSD11B2, CNKSR2, Rarb, and miR-195-x. The ceRNA network may offer valuable insights into the induction of primary hair follicle formation.

### Differential expression of ceRNA-RT-qPCR validation

**Fig. 7:**
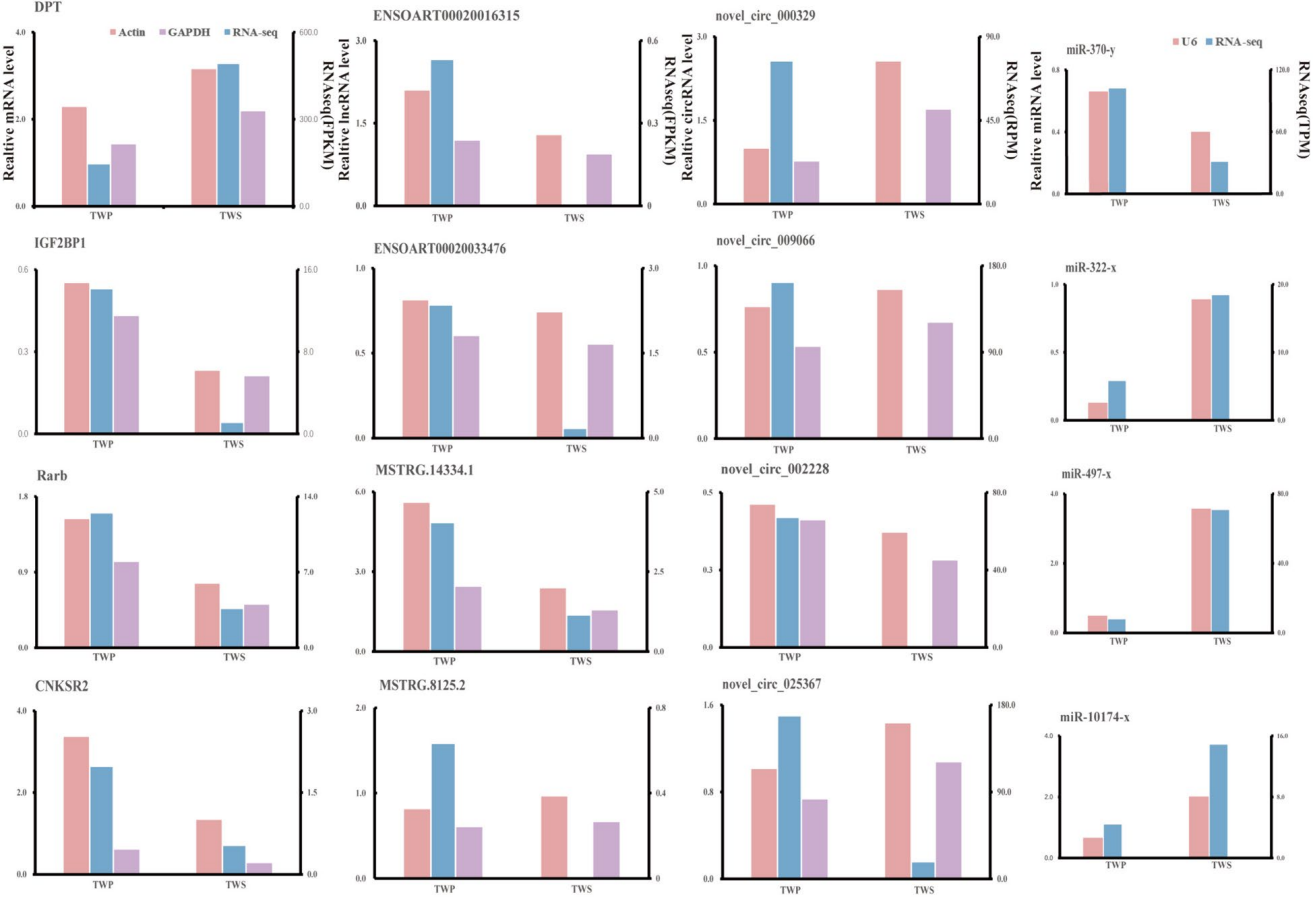
The expression level of four transcript types (mRNA, circRNA, lncRNA and miRNA) in the ceRNA network were validated by RT-qPCR. The left y-axis represents the RT-qPCR data, while the right side displays the RNA-seq data

The selection of four mRNAs (DPT, IGF2BP1, Rarb, and CNKSR2), four lncRNAs (ENSOART00020016315, ENSOART00020033476, MSTRG.14334.1 and MSTRG.8125.2), and four circRNAs (novel_circ_000329, novel_circ_009066, novel_circ_002228, and novel_circ_025367) and 4 miRNAs (miR-370-y, miR-322-x, miR-497-x, and miR-10,174-x) were conducted validate the sequencing results using real-time quantitative PCR (Fig. 7). The consistency between their expression trends and sequencing results suggests the robustness of the gene expression profile data obtained in this study.

## Discussion

The wool hair follicle serves as a unique and invaluable model for fundamental research in the life sciences, offering fertile ground to explore the underlying mechanisms of hair follicle morphogenesis, regeneration, heredity related to hair growth, and hair structure. The previous studies have demonstrated that during the induction stage of primary hair follicles, the regulatory mechanisms governing wool and hair follicle induction are largely conserved; however, distinct morphological changes occur with different characteristic genes, including certain regulatory elements such as lncRNA, which exhibit non-conserved functions across species [27, 28].

Although the role of lncRNA in hair follicle and skin development has been investigated, the precise mechanism underlying hair follicle induction remains elusive. Nie et al. identified 62 differentially expressed lncRNAs in primary hair follicles and predicted their potential targets, including Wnt16, Bmp3, and Itgb4, which are significantly enriched in key pathways such as WNT signaling, Hedgehog signaling, focal adhesion, and ECM receptor interaction. These findings align with our own study results and further underscore the crucial involvement of these pathways in hair follicle morphogenesis and skin development [14]. LNC_002919 and novel_circ_0026326 were found to act as ceRNA and participated in the regulation of the HF cycle as miR-320-3p sponges during skin development [29]. LncRNA MSTRG14109.1 and circRNA452 were competed with miRNA-2330 to regulated the cashmere fineness [30]. The findings suggest that ncRNAs may have a multifaceted and pivotal role in the regulation of hair genesis and growth. Furthermore, conducting a comprehensive analysis involving multiple regulatory elements could enhance our understanding of this intricate biological process. In this study, our conducted a comprehensive analysis of full transcriptome sequencing across different stages of hair follicle development, with the objective of identifying the genetic components involved in primary hair follicle occurrence and development. Currently, there is a lack of research on ceRNA network regulation pertaining to wool fiber characteristics in Tibetan sheep from Qinghai Province. Therefore, we first constructed the ceRNA regulatory network within the skin tissue of coarse wool fetal sheep.

A total of 58 DE lncRNAs, 325 circRNAs, 924 mRNAs, and 228 miRNAs were identified through high-throughput sequencing, playing a pivotal role in facilitating the development of primary hair follicles. The number of circRNAs annotated in primary hair follicles in this study was comparatively lower than the number annotated in secondary hair follicle studies conducted on cashmere goats (876 circRNAs) [31], and higher than the non-coding RNAs sequencing results were obtained between small waves and straight wool groups (114 circRNAs) [32]. However, this result is similar to the results of systematic analysis of non-coding RNAs in Angora rabbits during three hair follicle (HF) growth stages (247 circRNAs) [29]. In terms of the types and proportions of ncRNA sequencing, long intergenic noncoding and typical circRNAs accounted for the largest proportion, which was consistent with previous studies on sheep fetal and postnatal hair follicle development and adheres to established patterns [33, 34].

JAK-STAT, TGF-β, Hedgehog, PPAR, cGMP-PKG signaling pathway have been identified as crucial regulators in development of fibroblasts and epithelium during skin and hair follicle induction as well as hair follicle morphogenesis and skin development hair follicle induction stimulation [35–38]. In our data, these pathways mentioned exhibit a significant enrichment of differentially expressed genes in both mRNA and ncRNAs, forming a complex genetic regulatory network. This suggests that these signaling pathways may play a crucial role in regulating the growth of hair follicles. Additionally, we observed a significant enrichment of DEMs, DECs and DELs in the Focal adhesion and the ECM receptor interaction pathways, mirroring the enrichment findings in goats [39, 40]. The WNT signaling pathway is active in both the epithelial and mesenchymal components of hair follicle development, and WNT signaling is essential for promoting proliferation of the follicular epithelium and facilitating dermal condensation into dermal papillae, maintaining the properties necessary for hair follicle induction playing a crucial role in controlling hair follicle development and circulation [41–43]. Our analysis revealed a significant enrichment of DEGs in the WNT pathway, highlighting the crucial role played by the WNT pathway in primary hair follicle dermal condensation formation. Additionally, the circadian entrainment and glycosphingolipid biosynthesis signaling pathways play a pivotal role in the regulation of hair follicle regeneration [44, 45].

To further elucidate the key candidate genes underlying primary hair follicle induction, we conducted additional gene identification, integrated DEGs with DELs and DECs, and constructed a comprehensive circRNA/lncRNA–mRNA cross-regulatory network map. The presence of Dsc1 has been observed between cells at various stages of terminal differentiation in hair follicle (HF) keratinocytes [46], and knockout mice demonstrate epigenetic-follicular degeneration in older individuals [47]. The ALX4 gene plays a crucial role in the development of hair follicles, and individuals with homozygous c.793 C→T nonsense mutations in this gene may experience complete alopecia [48]. The gene HOXC13 may exert regulatory effects on hair follicle circulation in the context of LNC_000881 [49] and have been pinpointed as key determinants of wool fineness in sheep [50]. The protein KRT77, belonging to the type II keratin family, plays a crucial role in the formation of epidermis and coat formation [51]. Competitive adsorption of miR-184 liberates FGF10, which is implicated in hair follicle development, from chi-circRNA-0001141 [52]. Network analysis revealed the presence of genes such as TSPEAR [53], Egr3 [54], MEGF9 [55], SHH [56], PTGER2 [57], TGM3 [58], S100A7 [59], and SOX9 [60] that are intricately associated with hair follicle development and wool-related traits. The findings of these studies suggest that the screened wool-related traits candidate mRNAs and ncRNAs may unveil key factors involved in hair follicle growth, thereby serving as potential targets for subsequent investigations.

The regulation of targeted mRNAs expression by miRNAs governs a diverse array of cellular processes and functions, while also engaging in interactions with ncRNAs to form an intricate and interconnected ceRNA network, thereby playing a pivotal role in the regulation of target genes [61]. The expression and activity of the identified miR-195 [62], miR-370-3p [63], miR-432 [64], the miR-200 family [65], and miR-21 [66] can be regulated by target gene products. Changes in RNA abundance have the potential to impact ceRNA interactions [67], ultimately influencing hair follicle formation and development. Consequently, these findings suggest that candidate genes associated with various wool fiber traits are implicated in these molecular events. Our findings unveil several potential ceRNA regulatory networks involved in the process of hair follicle development and fiber growth. It has been reported that IGF2BP1 [68], HSD11B2 [69], TSPEAR [53], DPT [70], CNKSR2 [66], Rarb [71], and miR-195-x [62] play crucial roles in regulating hair follicle growth. Furthermore, the expression levels of IGF2BP1 [31], CNKSR2 [66] and Rarb [72] are intricately regulated through competitive binding interactions between lncRNAs/circRNAs and miRNAs during the process of hair follicle formation or signaling pathway activation. The reliability of gene expression in the ceRNA network was also confirmed by our RT-qPCR analysis. Apparently, the interaction between lncRNA/circRNA-mRNA and miRNA, as well as the targeting relationship between miRNA and mRNA in the ceRNA network, unequivocally underscores the pivotal role of miRNA in primary hair follicle and skin development of sheep, highlighting the intricate nature of the mechanism governing hair follicle initiation and development. Further investigations are required to elucidate the underlying mechanisms of action of these candidate mRNAs and non-coding RNAs in hair follicle induction, as well as to incorporate additional omics data (such as DNA methylome, ATAC-Seq, single-cell sequencing, and spatial transcriptomes) for the identification of molecular drivers involved in the regulation of hair follicle growth. In conclusion, a more comprehensive genomic data will enable us to gain a better understanding of the genetic basis underlying complex trait variation and pave the way for selective breeding strategies targeting wool traits in this particular breed.

## Conclusion

In summary, we employed whole-transcriptome sequencing technology for the first time to characterize the expression profiles of mRNAs, lncRNAs, circRNAs, and miRNAs during primary hair follicle development in. coarse sheep fetal skin. The present study unveils crucial molecular features underlying the primary hair follicle formation and underscores the intricate regulatory networks between coding and non-coding genes during hair follicle development. Additionally, it unveils the underlying molecular mechanisms governing the interplay between coding and non-coding RNAs, thereby providing a valuable resource for comprehending the biology of hair follicle development and unraveling the genetic foundation of wool-related traits. Moreover, it facilitates subsequent coupling of molecular element that drive their wool-related traits morphological manifestation.

### Supplementary Information

Supplementary Material 1.

## Data Availability

The raw reads produced in this study were deposited in the NCBI SRA with the accession number SRA SUB13911514 under Bio-project PRJNA1033028. Additional data can be found in supplementary files.

## Abbreviations

ncRNA: Non-coding RNA
circRNA: Circular RNA
lncRNA: Long non-coding RNAs
miRNA: Micro RNA
ciRNA: Circular intronic RNA
ceRNA: Competing endogenous RNAs
lincRNAs: long intergenic noncoding RNA
FPKM: fragments per kilobase per million mapped fragments
RPM: reads per million mapped reads
TPM: transcripts per million
GO: Gene Ontology
KEGG: Kyoto Encyclopedia of Genes and Genomes
PCC: Pearson correlation coefficient
SCC: Spearman Rank correlation coefficient
DEGs: differentially expressed genes
